# Machine Learning–Based Prediction of Long-Term Intraocular Pressure Fluctuations in Open-Angle Glaucoma

**DOI:** 10.1016/j.xops.2026.101240

**Published:** 2026-05-18

**Authors:** Colya N. Englisch, André M. Trouvain, Philip Wakili, Moritz J. Wolf, H. Burkhard Dick, Kaweh Mansouri, Esther M. Hoffmann, Marc J. Mackert, Achim Langenbucher, Jan Alexandersson, Karl T. Boden, Peter Szurman

**Affiliations:** 1 Eye Clinic Sulzbach, Knappschaft Hospitals Saar, Sulzbach/Saar, Germany; 2 Department of Experimental Ophthalmology, Saarland University, Homburg/Saar, Germany; 3 Saarland Informatics Campus, German Research Center for Artificial Intelligence (DFKI), Saarbrücken, Germany; 4 Knappschaftskrankenhaus Eye Clinic, University Hospital of the Ruhr University Bochum, Bochum, Germany; 5 Swiss Visio Glaucoma Research Centre, Montchoisi Clinic, Lausanne, Switzerland; 6 Department of Ophthalmology, University of Colorado, Denver, USA; 7 Department of Ophthalmology, University Medical Centre of the Johannes Gutenberg University Mainz, Mainz, Germany; 8 Department of Ophthalmology, LMU University Hospital, LMU Munich, Munich, Germany; 9 Klaus Heimann Eye Research Institute (KHERI), Sulzbach/Saar, Germany

**Keywords:** Glaucoma, Artificial intelligence, Machine learning, Intraocular pressure, Fluctuation

## Abstract

**Objective:**

To investigate the predictability of long-term intraocular pressure (IOP) fluctuations in open-angle glaucoma eyes implanted with a telemetric IOP sensor.

**Design:**

A prospective, open-label, single-arm, multicenter study.

**Subjects:**

Twenty-four patients were enrolled, including 20 with primary open-angle glaucoma, 2 with pseudoexfoliative, 1 with pigmentary, and 1 with uveitic glaucoma. Mean age was 65.2 ± 10.2 years.

**Methods:**

Telemetric IOP measurements were aggregated into nyctohemeral means. The first 90 postoperative days were excluded. A rolling reference framework was applied, in which temporally paired observations were generated by comparing each eligible day with a future time point at fixed prediction horizons. The relationship between short-term (7, 14, or 28 days) and long-term (273 or 364 days) fluctuations was assessed using Pearson correlation. Multivariate regression was applied to predict long-term fluctuations based on short-term data. In addition, supervised machine learning with a Random Forest Classifier was used to predict long-term fluctuations from clinical, demographic, and IOP-derived features. For each horizon (273 or 364 days) and threshold (+2.0, +3.0, and +4.0 mmHg), changes in mean nyctohemeral IOP were calculated. Outcomes were labeled as “1” if the increase met or exceeded the threshold and “0” otherwise.

**Main Outcome Measures:**

Predictability of long-term IOP fluctuations at 273 and 364 days.

**Results:**

Short-term fluctuations correlated only weakly with long-term variability (Pearson *r* ≤ 0.33) and explained at most 15.2% in regression analysis. Across 1224 Random Forest Classifier models, 47 met inclusion criteria of area under the receiver operating characteristic curve (AUROC) >0.8 and sensitivity and specificity >0.7 (27 for 364 days, 20 for 273 days). On average, 5563 ± 116 valid pairings from 9.2 ± 0.8 patients were used per configuration. Five final configurations were selected for each threshold–horizon combination based on the highest F1 values. Performance metrics included AUROC 0.81 to 0.86, cross-validated AUROC 0.78 to 0.83, accuracy 0.72 to 0.81, sensitivity 0.72 to 0.78, specificity 0.70 to 0.82, precision 0.32 to 0.44, and F1 value 0.44 to 0.56. All models included 7, 14, and 28-day fluctuations, mean nyctohemeral IOP, ocular pulse amplitude, age, body mass index, and central corneal thickness as predictors, with mean nyctohemeral IOP contributing most (38%–55%).

**Conclusions:**

Long-term IOP fluctuations can be predicted from baseline clinical and demographic data combined with IOP-related features. Telemetric devices and remote IOP monitoring, combined with predictive modeling, could reduce the burden of time-intensive procedures and health care costs while supporting individualized care in the face of rising demand.

**Financial Disclosure(s):**

Proprietary or commercial disclosure may be found in the Footnotes and Disclosures at the end of this article.

Elevated intraocular pressure (IOP) is the primary modifiable risk factor for glaucoma progression, a leading cause of irreversible blindness worldwide.^1^^,^^2^ Although the role of IOP fluctuations remains debated and requires further research, several studies have suggested an association with disease progression.^3^, ^4^, ^5^, ^6^, ^7^, ^8^, ^9^, ^10^, ^11^, ^12^, ^13^, ^14^, ^15^ Most of these fluctuations, ranging from instantaneous and nyctohemeral to short-term or long-term variations,^3^^,^^16^, ^17^, ^18^, ^19^, ^20^, ^21^, ^22^, ^23^ are inherently challenging to characterize, as meaningful assessment requires high-frequency measurements. While nyctohemeral fluctuations can be evaluated using repeated Goldmann applanation tonometry over a 24-hour period,^21^ high-frequency long-term monitoring has only recently become feasible with the advent of implantable telemetric IOP sensors, such as the EyeMate-SC.^22^^,^^23^

Despite rapid advances in telemetric sensor technology,^24^ its integration into routine clinical practice remains limited, often constrained by regulatory approval and reimbursement policies. Moreover, the implantation of IOP sensors may raise safety concerns, although no major adverse events have been reported to date in the first-in-human cohort of 24 patients supplied with the EyeMate-SC.^25^, ^26^, ^27^ Given that short-term IOP fluctuations are often assessable with conventional methods,^21^ a relevant clinical question emerges: can long-term IOP fluctuations be predicted based on short-term variability?

In this study, we aimed to examine the relationship between short-term and long-term IOP fluctuations, before evaluating the performance of multivariate regression models and of machine learning (ML) algorithms for predicting long-term IOP fluctuations.

## Methods

### Study Design and Subjects

This study is based on data from the 1-year prospective, open-label, single-arm, first-in-human, multicenter ARGOS-SC01 trial and its subsequent 2-year follow-up (ARGOS-SC01_FU).^25^, ^26^, ^27^ These studies showed a favorable safety and accuracy profile of the suprachoroidal EyeMate-SC (G-Metrics GmbH, previously Implandata Ophthalmic Products GmbH) implant in patients with glaucoma undergoing nonpenetrating glaucoma surgery (NPGS, i.e., canaloplasty and nonpenetrating deep sclerectomy).^25^, ^26^, ^27^ Twenty-four patients were enrolled, including 20 with primary open-angle glaucoma, 2 with pseudoexfoliative, 1 with pigmentary, and 1 with uveitic glaucoma. Details regarding exclusion criteria, implantation procedure, the device, its handling, and telemetric measurements have been described in previous publications.^25^, ^26^, ^27^, ^28^, ^29^, ^30^

Written informed consent was obtained from all participants. The study adhered to the Declaration of Helsinki and was approved by the local Institutional Review Board (Ethikkommission bei der Ärztekammer des Saarlandes, CIV-18-07-025065; Approval Number: 141/18; Date: October 30, 2018).

High-frequency telemetric IOP measurements were processed to derive nyctohemeral summary values (i.e., mean nyctohemeral IOP, including day and nighttime values) and short-term fluctuation features. These features were used to predict long-term IOP fluctuations over predefined prediction horizons using both statistical modeling and supervised ML approaches.

### Handling of Longitudinal Data and Discontinuities

To address clinically relevant discontinuities in longitudinal IOP time series, which may arise from additional interventions, medication changes, or prolonged gaps in follow-up, a structured preprocessing procedure was applied. To search for discontinuities in the IOP time series, mean nycrohemeral IOP values were smoothed using a 28-day forward and backward moving average, and time points were flagged as discontinuities when the difference between preceding and subsequent smoothed means exceeded 5 mmHg. In addition, gaps of >180 days between measurements were considered discontinuities, assuming potential intervening clinical changes.

Each patient time series was segmented into continuous intervals between such discontinuities. These segments were treated as independent analytical entities referred to as pseudopatients during model training. Transitional data points adjacent to discontinuities were excluded because they likely represented physiologically unstable periods that are not suitable for modeling.

### Definition of IOP Fluctuations

Mean nyctohemeral IOP was computed by averaging all measurements recorded within each day, thereby reducing circadian sampling bias, as previously described.^22^

**Figure 1:**
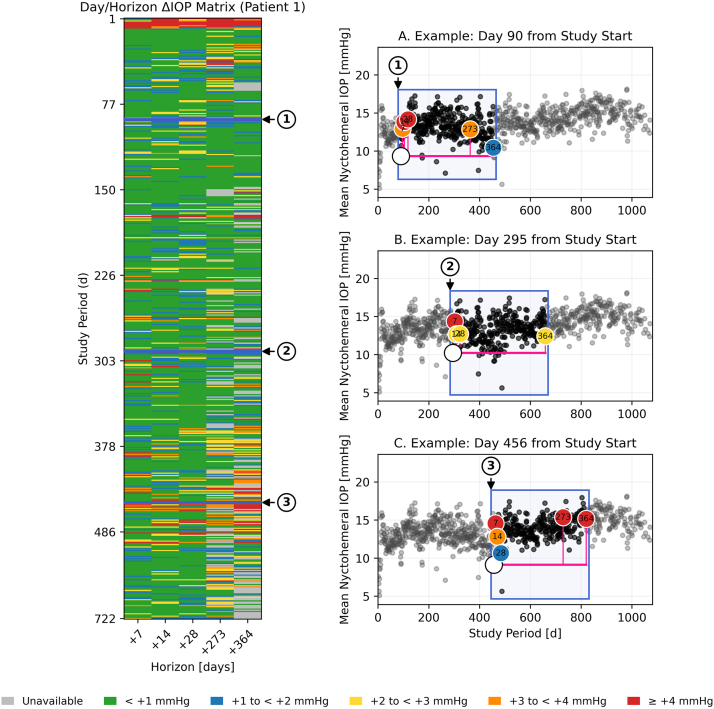
Transformation of longitudinal IOP data into fluctuation data. For a representative patient, nyctohemeral IOP measurements over ∼2 years are converted into paired ΔIOP values at predefined future horizons (+7 to +364 days). The heatmap (left) displays all eligible study days (rows) and corresponding ΔIOP values across time horizons (columns), with color indicating magnitude and gray denoting missing data. The panels **(A–C)** on the right show 3 examples of this process, illustrating how a selected start day is linked to future IOP measurements to derive ΔIOP. This procedure is repeated for all eligible days, generating a large set of paired comparisons that form the basis for subsequent predictive modeling. IOP = intraocular pressure.

Intraocular pressure fluctuations were assessed using a rolling framework across both short-term and long-term time scales. For each eligible day *x*, short-term changes were calculated as the difference between mean nyctohemeral IOP at day *x* and at day *x + 7, 14,* or *28*, when both time points were available and located within the same continuous segment, as illustrated in Figure 1.

Long-term fluctuations were defined analogously using prediction horizons of 273 and 364 days. All time intervals were restricted to multiples of 7 days to ensure consistent weekday alignment and to reduce potential bias related to weekday-dependent behavioral or measurement patterns.

All reported fluctuations are summarized as median ± interquartile range.

### Definition of Outcome and Prediction Framework

We formulated the prediction task as a binary classification problem to identify long-term IOP fluctuations. Outcomes were defined as fluctuations in mean nyctohemeral IOP over fixed prediction horizons of 273 and 364 days.

To maximize data utilization and avoid dependence on a single baseline, we applied aforementioned rolling reference framework. Analyses began on postoperative day 91, with the first 90 days excluded to mitigate early postoperative instability.

For each pair of day *x* and day *x + horizon* (ΔIOP), binary labels were generated according to predefined clinically relevant thresholds (+2.0, +3.0, and +4.0 mmHg). An outcome value of 1 indicated that the increase in IOP met or exceeded the specified threshold, while 0 indicated otherwise.

### Feature Construction

Predictive features were derived from IOP measurements as well as from clinical and demographic data. Intraocular pressure–related features included mean nyctohemeral IOP, ocular pulse amplitude (OPA), and short-term fluctuations at 7, 14, and 28 days. All fluctuation-based features were calculated relative to the same reference day *x* used for outcome definition.

Ocular pulse amplitude was estimated from the instantaneous variance of raw sensor signals. Each measurement provided by the telemetric sensor represents the mean of 10 submeasurements acquired over approximately 1 second. Ocular pulse amplitude corresponds to the variance of these underlying submeasurements and reflects cyclic IOP variation associated with the cardiac cycle. For each postoperative day, feature values were averaged across all measurements to obtain nyctohemeral summary metrics.

Clinical and demographic features included age, body mass index (BMI), central corneal thickness (CCT), and the number of prescribed ocular hypotensive medications. Categorical variables included sex and type of NPGS (canaloplasty vs. deep sclerectomy).

### Statistical Analysis

Correlations between short-term and long-term IOP fluctuations were assessed using Pearson product moment correlation coefficients. To further evaluate predictive relationships, multivariate logistic regression models were constructed to estimate long-term fluctuations over horizons of 273 and 364 days based on short-term fluctuations at 7, 14, and 28 days. Model fit was assessed using the coefficient of determination *R*^*2*^, representing the proportion of variance in long-term outcomes explained by short-term predictors.

### ML Model

To enhance predictive performance, a Random Forest Classifier (RFC) was applied as a supervised ML approach based on ensemble decision trees.^31^ Models were trained on structured feature sets comprising IOP-derived clinical and demographic variables. Alternative algorithms including CatBoost and XGBoost were evaluated during preliminary analyses but demonstrated inferior performance and were therefore not included in the final modeling framework.

### Model Training, Validation, and Overfitting Mitigation

Model performance was assessed using a patient-wise standard cross-validation strategy to ensure generalizability and to prevent data leakage.^32^ In this approach, all data from a given patient or pseudopatient segment were assigned exclusively to either the training or validation set. The dataset was partitioned into 5 folds such that each patient contributed to validation exactly once. For each fold, models were trained on the remaining data and evaluated on unseen patients. Predictions from all folds were aggregated to obtain a complete set of out-of-sample estimates.

To reduce overfitting, datasets with insufficient observations, defined as <250 or 300 pairings depending on the model, were excluded.

Feature reduction strategies were also explored. Automated selection methods did not improve performance, whereas manual selection guided by clinical reasoning enabled exclusion of noninformative variables and improved model robustness.

### Performance Metrics

Model performance was evaluated using multiple complementary metrics. Discriminative ability was assessed using the area under the receiver operating characteristic curve (AUROC) and its cross-validated counterpart (AUROC_CV_).^33^ Additional metrics included accuracy, defined as the overall proportion of correct predictions, sensitivity as the recall for outcome 1 and the proportion of true fluctuations correctly predicted, specificity as the recall for outcome 0 and the proportion of stable cases correctly predicted, precision as the proportion of predicted fluctuating cases that were correct, and the F1 score as the harmonic mean of sensitivity and precision.

Confusion matrices comprising true positives, true negatives, false positives, and false negatives were used to further characterize classification performance.

### Supervised Learning Framework and Model Selection

An automated batch processing framework was implemented to systematically explore combinations of modeling parameters, including different prediction horizons and thresholds, alternative strategies for handling discontinuities in patient timelines, varying minimum data requirements per segment, and different model configurations. Each configuration was evaluated using the performance metrics defined earlier. Only models achieving an AUROC >0.8 and both sensitivity and specificity >0.7 were retained. Among these, the configuration with the highest F1 score was selected for final reporting.

After evaluation, final models were retrained on the full dataset to derive feature importance measures.

### Software

All analyses were performed in Python (version 3.8.18, Python Software Foundation). Machine learning models and evaluation procedures were implemented using the Scikit Learn library (version 1.7.1).^34^

## Results

### Demographics, Ocular Characteristics, and Follow-Up Measurements

Half of the enrolled patients were female, and half were male. The mean age at surgery was 65.2 ± 10.2 years. Twelve eyes were right, and 12 were left. A total of 16 eyes were phakic, 8 were pseudophakic, and 2 phakic eyes underwent both phacoemulsification and canaloplasty. Overall, 15 eyes were treated with canaloplasty and 9 with deep sclerectomy. The median preoperative IOP was 19.3 ± 5.3 mmHg, with a range of 13.0–36.3 mmHg. Mean baseline CCT was 527 ± 41 μm. Twenty-two patients were White, while 1 was African, and 1 was Asian. Mean baseline BMI was 26.8 ± 5.2 kg/m^2^. Ocular hypotensive medication was taken, starting at postoperative day 90, in total on 4314 days, by 10 patients over 431.4 ± 367.3 days (range: 8–982), with an average of 1.2 ± 0.4 drugs per patient. At 360 days, 23 patients completed the visit, followed by 19 at 540 and 18 at 720, 19 at 900, and 18 at 1080 days. The follow-up duration lasted 944.1 ± 283.9 days. A total of 103 885 measurements were recorded. Of these, 91 987 were recorded after the early postoperative period of 90 days (midnight–2:59 am: 1365 [1.5%]; 3–5:59 am: 615 [0.7%]; 6–8:59 am: 19 063 [20.7%]; 9–11:59 am: 14 213 [15.5%]; noon–2:59 pm: 16 251 [17.7%]; 3–5:59 pm: 14 310 [15.6%]; 6–8:59 pm: 13 218 [14.4%]; 9 pm–midnight: 12 952 [14.1%]). On average, 3832.8 ± 3413.8 IOP readings per patient were achieved. Excluding measurement-free days, the IOP reads per patient amounted in average 5.12 ± 3.30 daily.

### Short-Term IOP Variability Weakly Predicts Long-Term Fluctuations in Multivariate Logistic Regression

**Figure 2:**
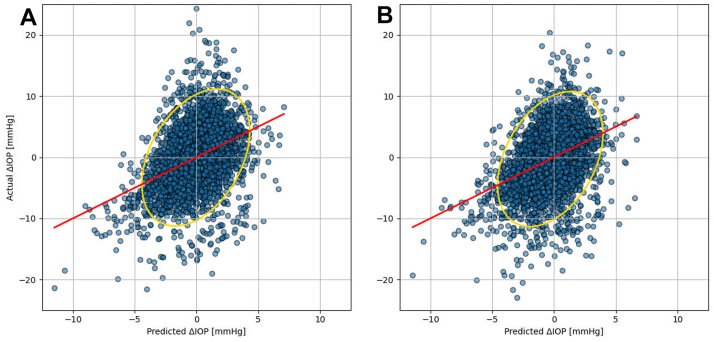
Predicted vs. actual IOP fluctuations (ΔIOP) from multivariate logistic regression models estimating long-term variability at 273 days **(A)** and 364 days **(B)**. Each symbol represents an individual prediction, the red line indicates the regression fit, and the yellow ellipse denotes the 95% confidence region. IOP = intraocular pressure.

For Pearson product–moment correlation coefficients analysis, 30 070, 29 204, 28 184, 16 787, and 14 292 IOP pairings were available for the short-term intervals at 7, 14, and 28 days, and for the long-term intervals at 273 and 364 days, respectively. Short-term fluctuations correlated only weakly with long-term fluctuations (Pearson *r* ≤ 0.33, Table 1), and multivariate logistic regression showed they explained at most 15.2% of subsequent long-term variability (Fig 2, Table 2).

**Table 1:** Pearson Product–Moment Correlation Coefficients between Intraocular Pressure (IOP) Fluctuations within 7, 14, and 28 Days and Subsequent IOP Fluctuations at 273 and 364 Days

Fluctuation Intervals (Days)	7	14	28	273
14	0.53	–	–	–
28	0.49	0.53	–	–
273	0.33	0.33	0.30	–
364	0.31	0.33	0.31	0.60

**Table 2:** Intercepts, Parameter Estimates for Short-Term IOP Fluctuations at 7, 14, and 28 Days, and *R*^*2*^ Values from Multivariate Logistic Regression Models Predicting Long-Term IOP Fluctuations at 273 and 364 Days

Horizon	ϐ_7_	ϐ_14_	ϐ_28_	Intercept	*R*^*2*^
273	0.277	0.250	0.180	–0.0495	0.152
364	0.232	0.261	0.196	–0.2989	0.149

IOP = intraocular pressure.

### RFC Reliably Predicts Long-Term Fluctuations

**Figure 3:**
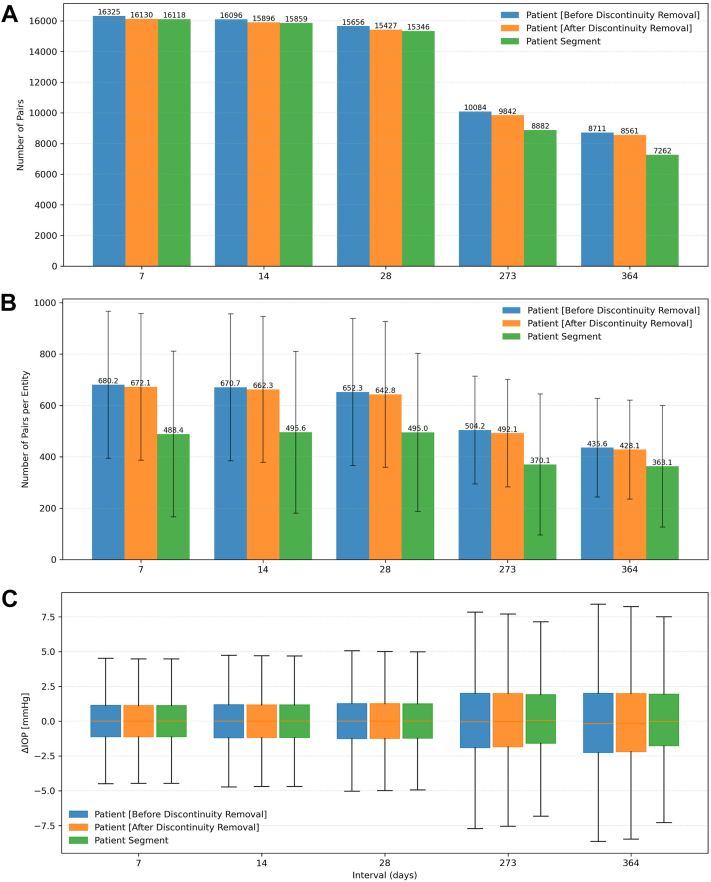
Fluctuation pairings across intervals of 7, 14, 28, 273, and 364 days. **A,** Total number of fluctuation pairs per patient before (blue) and after (orange) discontinuity removal, as well as per patient segment (green). **B,** Mean ± SD of fluctuation pairs per entity (patient before/after discontinuity removal, and patient segment). **C,** Intraocular pressure fluctuation data across the same intervals: red line = median, box = interquartile range (IQR), whiskers = 1.5 × IQR; outliers not shown. IOP = intraocular pressure; SD = standard deviation.

Figure 3 shows raw fluctuation data: the total number of fluctuation pairings per interval (Fig 3A), per interval and per entity (i.e., per patient before or after discontinuity removal and per patient segment; Fig 3B), and the actual fluctuation values (Fig 3C). Mean nyctohemeral IOP was 11.4 ± 3.9 mmHg between days 90 and 180, 11.1 ± 3.5 mmHg between days 180 and 360, 11.5 ± 3.8 mmHg between days 360 and 540, 10.6 ± 3.2 mmHg between days 540 and 720, 10.3 ± 3.1 mmHg between days 720 and 900, and 10.4 ± 2.9 mmHg between days 900 and 1080.

Overall, in 6 patients, no valid pairings (i.e., without values for all short-term fluctuations and the investigated horizon) were available for either long-term horizon. After exclusion, the mean number of valid pairings per patient was 442.6 ± 222.5 for the 273-day horizon and 389.8 ± 201.8 for the 364-day horizon. For both horizons, 11 patients had >250 valid pairings and 10 had >300.

Across both horizons, 52 patient segments were considered. For the 273-day horizon, 31 segments yielded no valid pairings; for the 364-day horizon, 34 segments. After exclusion, the mean number of valid pairings per patient segment was 351.6 ± 266.7 (n = 21) for the 273-day horizon and 344.3 ± 230.7 (n = 18) for the 364-day horizon. For both horizons, 9 segments had >250 valid pairings and 8 had >300. None of the segments stem from the same patient.

A total of 1224 ML models were trained across the full range of input parameter variations. Inclusion criteria of AUROC >0.8 and sensitivity and specificity >0.7 were met in 47 models: 27 predicting the 364-day horizon and 20 predicting the 273-day horizon. A threshold of 2 mmHg was met in only 2 models, both for the 273-day horizon. A threshold of 3 mmHg was met in 30 models and of 4 mmHg in 15 models. Models for the 273-day horizon used segmentations after discontinuity removal, whereas 364-day models used complete patient datasets. On average, 5563 ± 116.0 valid pairings from 9.2 ± 0.8 patients were used per configuration, with a minimum number of pairings of 300, except for 273-day predictions with thresholds of 3 and 4 mmHg (250).

**Figure 4:**
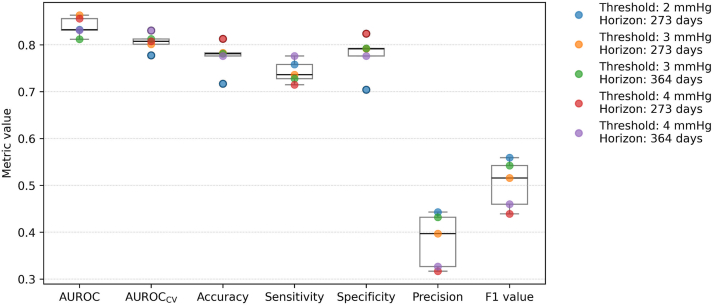
Metric values for the 5 random forest models with the highest F1 scores among those achieving AUROC >0.8 and sensitivity and specificity >0.7. Displayed metrics include AUROC, fivefold AUROC_CV_, accuracy, sensitivity, specificity, precision, and F1 score. In the boxplots, the middle line represents the median, the box the interquartile range (IQR), and the whiskers 1.5 × IQR. AUROC = area under the receiver operating characteristic curve; AUROC_CV_ = cross-validated area under the receiver operating characteristic curve.

**Figure 5:**
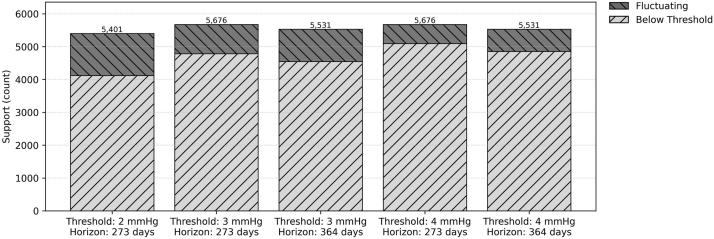
Column graphs showing the number of pairings predicted as fluctuating versus stable for each of the 5 model configurations.

**Figure 6:**
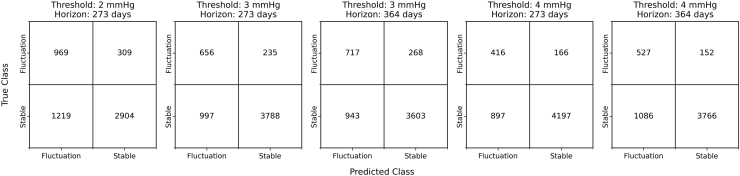
Confusion matrices for the top 5 performing threshold–horizon combinations.

For each threshold–horizon combination, the configuration with the highest F1 value was selected, resulting in 5 final configurations. The AUROC ranged from 0.81 to 0.86, AUROC_CV_ from 0.78 to 0.83, accuracy from 0.72 to 0.81, sensitivity from 0.72 to 0.78, specificity from 0.70 to 0.82, precision from 0.32 to 0.44, and F1 value from 0.44 to 0.56 (Fig 4). Figure 5 shows the number of pairings predicted as fluctuating (1) versus stable (0) for each of the 5 model configurations. Figure 6 shows the confusion matrices for the top 5 performing threshold–horizon combinations.

**Figure 7:**
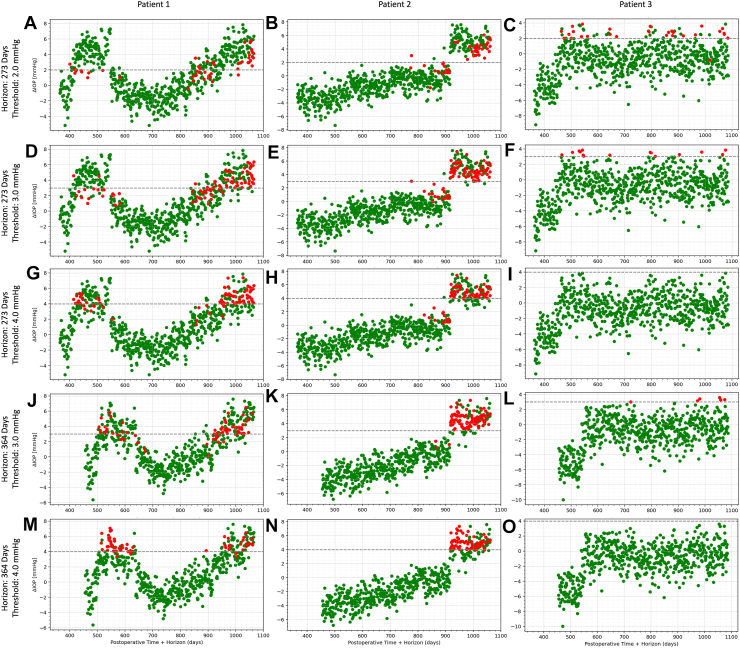
Application of the 5 random forest classifier models to 3 patients who contributed data to model training. Green indicates correctly predicted pairings, and red indicates incorrectly predicted pairings. Data from the 3 patients are arranged as follows: (**A**, **D**, **G**, **J**, and **M**), (**B**, **E**, **H**, **K**, and **N**), and (**C**, **F**, **I**, **L**, and **O**). Panels **(A–C)**, **(D–F)**, and **(G–I)** correspond to the 273-day prediction horizon, while panels **(J–L)** and **(M–O)** correspond to the 364-day horizon. IOP = intraocular pressure.

**Figure 8:**
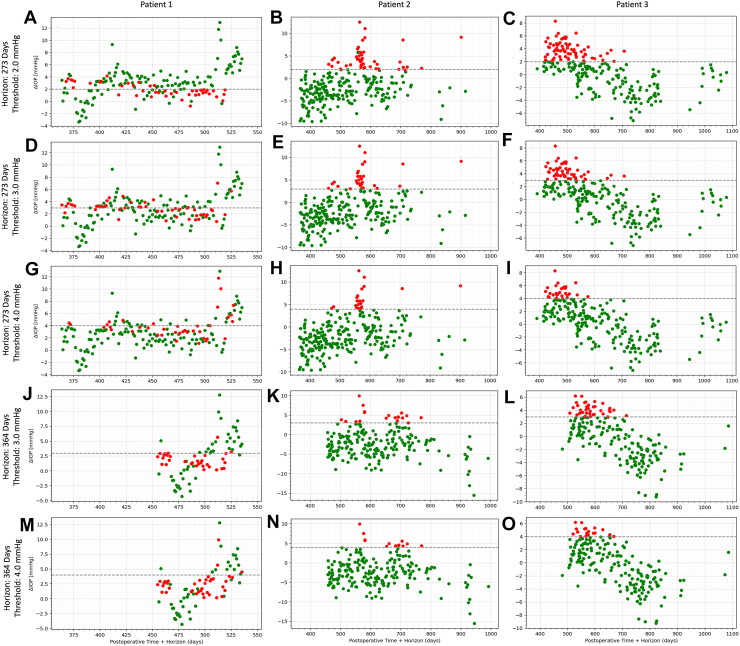
Application of the 5 random forest classifier models to 3 patients who did not contribute data to model training. Green indicates correctly predicted pairings, and red indicates incorrectly predicted pairings. Data from the 3 patients are arranged as follows: (**A**, **D**, **G**, **J**, and **M**), (**B**, **E**, **H**, **K**, and **N**), and (**C**, **F**, **I**, **L**, and **O**). Panels **(A–C)**, **(D–F)**, and **(G–I)** correspond to the 273-day prediction horizon, while panels **(J–L)** and **(M–O)** correspond to the 364-day horizon. IOP = intraocular pressure.

Figures 7 and 8 show applications of the 5 models on patients, 3 that contributed to the models (Fig 7), and 3 that were not included owing to insufficient data (<250 or 300 valid pairings; Fig 8).

**Figure 9:**
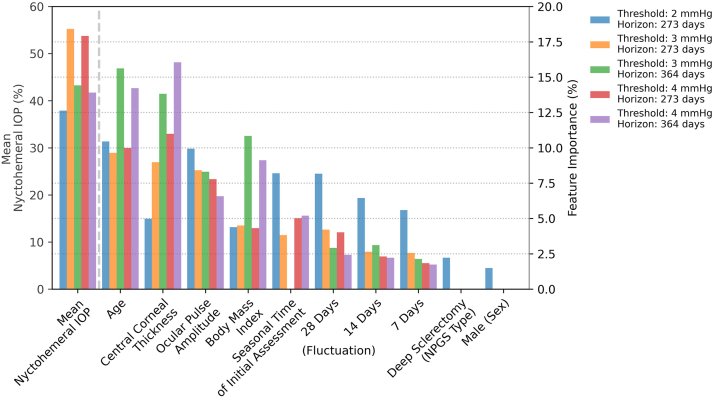
Feature importances (%) for the 5 random forest classifier models predicting long-term IOP fluctuations beyond the defined threshold. The left y-axis shows the percentage for mean nyctohemeral IOP, while the right y-axis shows the percentages for the remaining parameters, including short-term fluctuations at 7, 14, and 28 days, ocular pulse amplitude, seasonal timing of initial assessment, age, body mass index, and central corneal thickness (continuous variables), as well as sex (male) and NPGS (deep sclerectomy) as categorical variables. IOP = intraocular pressure; NPGS = nonpenetrating glaucoma surgery.

All 5 models included 7, 14, and 28-day fluctuation, mean nyctohemeral IOP, OPA, age, BMI, and CCT as features. Four models additionally included seasonal time of initial assessment, 2 included medication count, and 1 (273-day, 2 mmHg threshold) included categorical features NPGS type (deep sclerectomy) and sex (male). Mean nyctohemeral IOP was the most important predictor ranging from 38% to 55%. Figure 9 depicts the feature importance profiles for each configuration. Notably, short-term fluctuations contributed <10% to feature importance.

## Discussion

This study aimed, for the first time to the authors’ knowledge, to assess the predictability of long-term IOP fluctuations based on large amounts of telemetric data. While long-term fluctuations were poorly predicted by short-term fluctuations in multivariate logistic regression, we achieved strong predictive performance by incorporating clinical, demographic, and additional IOP-related input features using the RFC as ML algorithm.

All RFC models were trained using a supervised ML approach, pairing input features with binary outcome labels to indicate the occurrence of clinically relevant IOP fluctuations. To ensure realistic evaluation, we applied patient-level performance estimation, mimicking clinical routine deployment where predictions must generalize to unseen patients. This strategy effectively prevented data leakage but added complexity, especially for rare outcomes and sparsely represented categorical subgroups. Overall, confusion matrices showed that static progression is predicted more reliably than fluctuation, and a substantial proportion of static progression is misclassified as fluctuation. The number of these false positives exceeds the correctly classified fluctuation cases by approximately one-third to one-half. Taken together, this can be interpreted as evidence that the model has learned meaningful patterns. The elevated false-positive rate is likely attributable to class imbalance and the relatively small number of patients.

Previous studies have demonstrated that 24-hour IOP variations can be predicted from daytime IOP measurements. For instance, in a study involving 956 eyes undergoing 24-hour IOP monitoring, the XGBoost algorithm achieved an accuracy of 0.89, a specificity of 0.97, a sensitivity of 0.56, and an area under the curve of 0.89.^35^ Another study by the same group used random forest regression with IOP, sex, age, central corneal thickness, BMI, blood pressure, ocular perfusion pressure, spherical equivalent, and ocular hypotensive medication drug use as input parameters to predict 24-hour peak and average IOP.^36^^,^^37^ Overall, ML algorithms seem to be superior to regression methods as they are capable of apprehending complex nonlinear relationships.^36^, ^37^, ^38^, ^39^

These findings are particularly relevant for improving IOP monitoring, given the very limited availability of high-frequency measurements in routine clinical practice. Currently, assessment of nyctohemeral IOP profiles often requires 24–48-hour inpatient monitoring using Goldmann applanation tonometry, which is resource-intensive, costly, and burdensome for both patients and health care systems.

In contrast, our study utilizes high-frequency IOP data obtained from an intraocular telemetric sensor. Although implantation is invasive and associated with additional costs, it is performed during a medically indicated procedure and enables high-resolution, long-term IOP monitoring with fewer observer and timing-related biases than Goldmann applanation tonometry.^22^^,^^26^^,^^27^ Given the limited repeatability of manually acquired nyctohemeral IOP profiles,^40^ telemetric monitoring may offer a substantial advantage. Patients can perform multiple measurements throughout the day, generating detailed IOP curves that can be transmitted remotely, thereby facilitating timely and individualized treatment adjustments.

Alternative remote monitoring approaches exist but remain limited. Home rebound tonometry depends on patient adherence and is less suitable for nocturnal measurements, although it does not require surgical implantation; however, recurring costs for disposable components may be significant.^41^, ^42^, ^43^ Contact lens–based sensors, such as the Sensimed Triggerfish (Sensimed SA), provide 24-hour recordings but are costly, limited to short-term use, and report IOP-related changes in arbitrary units rather than mmHg.^9^^,^^44^

While the telemetric system used in this study was restricted to patient-initiated measurements, newer device iterations already enable automated and thus even more frequent data acquisition, for example, through wearable reader systems integrated into eyeglasses. Such truly near-continuous monitoring with hundreds to even thousands measurements per day is likely to provide deeper insights into the still-debated role of IOP fluctuations.

With the anticipated broader clinical adoption of telemetric technologies, the need to estimate short-term IOP profiles from sparse measurements may diminish. Instead, predicting long-term IOP patterns and fluctuations may become more clinically relevant, as this could enable earlier therapeutic intervention before structural damage or visual field loss occurs.

Regarding feature importance, mean nyctohemeral IOP was the top contributor, accounting for 38% to 55% in this study. However, while defining the main outcome as the difference between 2 time points was necessary to capture true long-term fluctuation, this approach introduces coupling that may artificially inflate the apparent feature importance of mean nyctohemeral IOP, as it directly contributes to the calculated fluctuation. Other relevant features included short-term fluctuations, OPA, seasonal timing of the initial assessment, BMI, age, and CCT. Categorical variables (i.e., sex and NPGS type) contributed minimally. The observed limited relevance of short-term fluctuations in ML predictions is consistent with the correlation and multivariate regression analyses. Notably, Rabiolo et al suggested that OPA may be an independent risk factor for glaucoma progression.^45^ Interestingly, baseline variables such as age, BMI, and CCT became increasingly important at higher thresholds and longer prediction horizons, with BMI known to be associated with IOP.^46^ Although within-day standard deviation was initially considered as a marker of short-term IOP variability, it was excluded from the final models due to its detrimental effect on performance, largely because days with only a single measurement had to be omitted. With larger datasets and systematic handling of days with insufficient measurements, within-day standard deviation or similar metrics may yet prove valuable for predicting long-term fluctuations.

Our study has several limitations, including a small sample size, potential bias from including eyes with low fluctuation profiles after NPGS, and variability in measurement timing due to patient-directed recording. While the models achieved excellent AUROC values, precision was limited (0.32–0.44), reflecting aforementioned small sample size.^31^^,^^47^ Unmeasured confounders such as lifestyle changes, hormonal fluctuations, and seasonal IOP variation may also have influenced results.^48^, ^49^, ^50^

It is important to emphasize the clinical relevance of these findings. In fact, the study is limited primarily and essentially by the low number of patients rather than insufficient data per patient. Despite this, it demonstrates a proof-of-concept: long-term IOP fluctuations can be predicted using ML algorithms based solely on data obtainable within a few weeks, at least in post-NPGS eyes. Future studies should assess predictability in larger and diverse patient populations, particularly those with larger IOP fluctuations. The use of telemetric sensors is important, as large patient numbers with few measurements per patient as could be recorded with standard routine methods would introduce other limitations.

Looking forward, wider implementation of IOP sensors could support individualized medicine and telemedicine. One promising application is the development of multivariate patient-specific nomograms, allowing clinicians to input baseline clinical, demographic, and additional IOP-related features for specific subgroups categorized according to diagnosis (e.g., primary open-angle glaucoma or pseudoexfoliation glaucoma) and surgery type (e.g., penetrating or nonpenetrating surgery), for instance, to predict long-term IOP trajectories. This could guide early treatment escalation before functional or structural deficits are observed.

In conclusion, long-term IOP fluctuations can be reliably predicted using baseline clinical and demographic variables together with IOP-related features such as mean nyctohemeral IOP, OPA, and short-term fluctuations. The main limitation of our study lies in the small patient sample, and these findings must therefore be validated in larger cohorts. If confirmed, this approach holds considerable potential for improving glaucoma management. Through the use of telemetric devices and remote IOP monitoring, predictive modeling could help reduce the burden of time-intensive procedures and health care costs, while addressing the growing demand–supply imbalance in glaucoma care.

## Declaration of Generative AI and AI-Assisted Technologies in the Writing Process

During the preparation of this work the authors used Chat Generative Pre-trained Transformer (GPT-5.2, OpenAI) for English language polishing. After using this tool, the authors reviewed and edited the content as needed and take full responsibility for the content of the published article.
